# Erector spinae plane block for postoperative analgesia for above-the-knee amputation: a case report

**DOI:** 10.1186/s13741-022-00271-2

**Published:** 2022-07-29

**Authors:** Erica M. Langnas, Andrew Gray, Matthias Braehler

**Affiliations:** 1 Department of Anesthesia and Perioperative Care, University of California, 8 Buchanan Street, Unit 808, San Francisco, CA 94102 USA; 2 Department of Anesthesia and Perioperative Care, Zuckerberg San Francisco General Hospital and Trauma Center, University of California, 8 Buchanan Street, Unit 808, San Francisco, CA 94102 USA

**Keywords:** Regional anesthesia, Erector spinae plane block, Above-the-knee amputation, Postoperative pain

## Abstract

**Background:**

Above-the-knee amputations (AKA) are common surgeries that frequently use neuraxial or peripheral nerve blocking techniques for both intraoperative and postoperative analgesia. It is not uncommon that patients present with contraindications to neuraxial anesthesia.

**Case presentation:**

We identified a relatively novel use of erector spinae plane block (ESP) for above-the-knee amputation that allows for adequate pain control postoperatively when there are contraindications for neuraxial.

**Conclusion:**

While data on ESP at the thoracic level is well described, less is known about the expected coverage for lumbar ESP. This case suggests that at the level of L3, there is sufficient dermatomal spread for an AKA.

## Introduction

Above-the-knee amputations (AKA) are common surgeries that frequently use neuraxial or peripheral nerve blocking techniques for both intraoperative and postoperative analgesia. We aim to add to the limited existing literature that erector spinae plane blocks (ESP) may be utilized for above-the-knee amputation to reduce postoperative pain in patients who may have contraindications to neuraxial or peripheral nerve blocks (Ayub et al. 2019; Balaban and Aydın 2019). ESP blocks are relatively novel and have been gaining popularity and proving effective for postoperative pain for many surgeries (Forero et al. 2016; Chin et al. 2017; Tulgar and Senturk 2018; Harbell et al. 2020). This case adds clinical evidence to the limited existing literature surrounding expectations of craniocaudal spread (Harbell et al. 2020).

Common regional anesthesia considerations for AKA are epidural, lumbar plexus, and peripheral nerve blocks. Epidural catheters may be used for surgical anesthesia and continued in the postoperative period. Limitations of epidurals are largely surrounding the inability to safely place them. Contraindications include patients with coagulopathy, receiving anticoagulation, and infection at the insertion site. Lumbar plexus blocks may be used for intra- and postoperative pain control. Limitations of this block are similar to those for an epidural catheter in regard to anticoagulation given the depth of the block as well as its proximity to the central neural structures (Horlocker et al. 2018). Femoral nerve block or fascia iliaca block alone or in conjunction with sciatic nerve block may be used for postoperative pain control. Limitations include potential sparing of the obturator nerve, resulting in inferior pain control. The patient provided written HIPAA authorization for details of the case to be published.

## Case presentation

We describe the case of a 74-year-old man who was scheduled for an urgent AKA. The patient was urgently admitted from home after his home nurse noticed a dehiscence of his prior right below-the-knee amputation (BKA) stump. He received his BKA surgery 1 month prior for critical limb ischemia due to vascular disease which was complicated by poor wound healing. During this prior admission for a BKA, he also received a right-sided common femoral endarterectomy procedure. His past medical history is significant for end-stage renal disease status post-transplantation, aortic stenosis status post-transcatheter aortic valve replacement, and atrial fibrillation for which he is on apixaban, heart failure with reduced ejection fraction, and peripheral vascular disease. Due to the concern for wound dehiscence and infection noted by his home nurse, he was admitted urgently, started on broad-spectrum antibiotics, and scheduled for an AKA. Given the likelihood of surgery, his apixaban was held on admission, and he was transitioned to a heparin drip. His heparin drip was titrated to a goal-activated partial thromboplastin time (aPTT) of 60–80 s (reference range 20.9–30.9).

On the morning of surgery, his heparin drip was stopped 4 h prior to surgery, and an aPTT was measured preoperatively which returned normal. Given the need for anticoagulation after the surgery and concerns for the difficulty of placement given the patient body habitus with a BMI of 41, neuraxial was avoided and the anesthesia team planned to proceed with a peripheral nerve block. At the request of the surgeons, there would be no catheter placement. In preparation for the femoral nerve block, it was noted that the site of his prior right common femoral endarterectomy appeared erythematous and concerning for soft tissue infection. As a result, the femoral nerve block was aborted. Given these limitations, it was decided to place a right-sided erector spinae plane block in the lumbar region. The patient was then positioned on his left side, and a preliminary scan was completed at the third lumbar space (L3). This level was chosen for two reasons. First, we expected spread one to two levels above and below along and second at this level we encountered the best ultrasound image quality. Midline, iliac crest, and the right transverse process were identified at L3. Preparation of the field with sterile preparation was completed. A 10-mm 21-gauge needle was inserted under ultrasound guidance. Gentle contact was made with the transverse process. Aspiration was negative, and 30 ml of 0.2% ropivacaine was injected under direct visualization. The local anesthetic spread was noted below the erector spinae muscle.

Intraoperatively, the patient received general anesthesia. Induction medications included 50 mg of propofol, 100 μg of fentanyl, 50 mg of rocuronium, and 60 ml of lidocaine. General anesthesia was maintained with sevoflurane between 1.3 and 2.0%, he received 2 g of cefazolin for antibiotics, and he received a total of 1 ml of hydromorphone IV in incremental doses over the last hour of the case. In addition, he received 4 mg ondansetron and 96 mg of sugammadex at the end of the case. He was extubated and taken to the post-anesthesia care unit without complications.

While in the PACU, the patient’s pain was monitored using a Numerical Rating Scale from 0 to 10 (0 = no pain, 10 = worst imaginable pain). His first three documented pain score levels were zero and over the next 3 h increased to a maximum pain score of 4 for which he received 1 g of acetaminophen and 5 mg of oxycodone. These are commonly provided pain medications for moderate pain in our PACU. He recovered appropriately and was transferred to the ward. The patient reported pain scores of 0 to 4 for 11 h postoperatively. On hour 12, his surgical site pain increased to 7, requiring the use of 0.4 mg hydromorphone IV and 5 mg of oxycodone. We hypothesize this change in pain occurred as the nerve block wore off. On postoperative day 1, the patient was interviewed about his experience with the nerve block. Consistent with the pain scores documented, he reported adequate pain control for about 10–12 h and then an abrupt change as the nerve block wore off.

## Discussion

It is not uncommon that patients present with contraindications to neuraxial anesthesia. Here, we identified a relatively novel use of ESP for above-the-knee amputation that allows for adequate pain control postoperatively. While data on ESP at the thoracic level is well described, less is known about the expected coverage for lumbar ESP. This case suggests that at the level of L3, there is sufficient dermatomal spread for an AKA.

Compared to femoral nerve block and fascia iliaca block, which do not necessarily cover the obturator nerve, the lumbar ESPB causes the spread of local anesthetic to the roots of the lumbar plexus, which includes the obturator nerve. This is critical as the obturator nerve innervates a major part of the femur which contributes to postoperative pain (Gadsden and Warlick 2015). While a lumbar plexus block could be performed, there are concerns regarding anticoagulation given the depth of the block and its close proximity to neuraxial structures. The lumbar ESPB, however, is considered a more superficial block, and there is emerging literature deeming it safe to perform this block using less strict guidelines compared with neuraxial and lumbar plexus blocks (Adhikary et al. 2019).

Beyond the perioperative period, AKA surgery is associated with post-amputation pain or phantom limb pain (Subramaniam et al. 2005). This type of pain results in significant morbidity and remains challenging to treat. The etiology is complex, and strategies to prevent or reduce the likelihood of post-amputation continue to be explored. It is unknown if lumbar ESP catheters reduce post-amputation pain, and this may represent a novel area for future studies. In addition, we described in our case discomfort from the surgical team in placing a catheter given anticoagulation requirements for the patient. When new blocks emerge, there may be discomfort with surgical teams surrounding catheter placement. We encourage dialogue with surgical teams surrounding the current literature and guidelines and the potential benefits of ESP blocks and how they compare with other common regional or neuraxial techniques.

In summary, this retrospective case report adds to relatively limited existing literature surrounding the potential uses of a lumbar ESP block. It is critical to increase the body of evidence on ESP blocks for orthopedic surgeries as this may result in a more rigorous evaluation of the ESP block along with studies that compare the effectiveness of ESP with other blocks.

## Data Availability

Data sharing is not applicable to this article as no datasets were generated or analyzed during the current study.

## Abbreviations

AKA: Above-the-knee amputation
ESP: Erector spinae plane
BKA: Below-the-knee amputation
L3: Third lumbar space
